# The COVID-19 Pandemic and Elective Spine Surgery—A Single Center Experience

**DOI:** 10.3390/medicina59091575

**Published:** 2023-08-30

**Authors:** Nenad Koruga, Anamarija Soldo Koruga, Silva Butković Soldo, Robert Rončević, Tatjana Rotim, Tajana Turk, Domagoj Kretić, Sonja Škiljić, Nenad Nešković, Alen Rončević

**Affiliations:** 1 Department of Neurosurgery, University Hospital Center Osijek, 31000 Osijek, Croatia; 2 Faculty of Medicine, Josip Juraj Strossmayer University of Osijek, 31000 Osijek, Croatia; 3 Department of Neurology, University Hospital Center Osijek, 31000 Osijek, Croatia; 4 Department of Diagnostic and Interventional Radiology, University Hospital Center Osijek, 31000 Osijek, Croatia; 5 Department of Anesthesiology and Critical Care, University Hospital Center Osijek, 31000 Osijek, Croatia

**Keywords:** body mass index, COVID-19, disc herniation, low back pain, overweight, pandemic, spinal stenosis, spine surgery

## Abstract

*Background and objective*: The COVID-19 pandemic had a profound impact on medical practice worldwide. In this study, we aimed to investigate the trends of elective spine surgery in our department before and during the pandemic. *Materials and methods*: Total number of spine procedures due to disc herniation (DH) or spinal stenosis (SS) was collected during 2019–2021 in the Department of Neurosurgery, University Hospital Center Osijek, Croatia. In order to elucidate potential risk factors in the post-pandemic period, demographic data were collected for patients who underwent surgery during 2021. *Results*: In 2020, there was a 22.1% decrease in the number of surgeries compared to 2019 (205 vs. 263), but during 2021 we observed an increase of 36.1% compared to 2020 (279 vs. 205). The mean age of patients in 2021 was 53.14 years (53.14 ± 13.05) with body mass index of 28.31 kg/m^2^ (28.31 ± 4.89). There were 179 overweight patients (74%) and 103 smokers (42.6%). Although male and female patients were equally represented (121 each), there was a significant interaction of weight class and sex (*p* = 0.013). Patients younger than 65 were more likely to undergo surgery due to DH (*p* < 0.001), whereas older patients were more likely to suffer from SS (*p* < 0.001). *Conclusions*: The volume of elective spine surgeries decreased in the first year of the pandemic and increased the following year. Our results suggest that public health policies in the early pandemic period reduced elective surgical procedures, which was followed by a compensatory increase in the following period.

## 1. Introduction

The COVID-19 pandemic had an enormous impact on our lives. In these unique circumstances, many individuals worldwide developed a more sedentary lifestyle, signified by a decrease in daily physical activity [1], and, at the same time, an increase in food intake, particularly of more unhealthy food options [2]. Consequently, these behavioral changes resulted in higher incidence and intensity of low back pain (LBP) all over the world [3]. LBP is one of the most common causes of emergency departments visits [4] and is the dominant cause of disability worldwide [5]. The pathophysiology of LBP is complex and is influenced by biological, social, and psychological factors [6]. Having increased body mass index (BMI) increases the risk of developing LBP—a finding which has been replicated in several studies [7,8,9,10]. BMI is a simple metric used to determine the weight status of an individual. Despite its demonstrated usefulness on a population-level, shortcomings of BMI on an individual level are worth noting [11]. The World Health Organization (WHO) has defined overweight as BMI greater than or equal to 25 kg/m^2^ and obesity as BMI greater than or equal to 30 kg/m^2^ [12]. However, correlation of LBP and excess body weight could be bidirectional. Being overweight or obese could render one more prone to LBP, but at the same time suffering from LBP reduces physical activity of an individual and increases body weight [7]. LBP is initially treated with noninvasive approaches, but if these fail to reduce pain, invasive approaches, such as spine surgery, are considered [13,14]. Generally, there are two main surgical methods for treating LBP based on clinical presentation and its correlation to radiologic findings. Patients presenting with disc herniation (DH) are subjected to surgical removal of the herniated disc, also known as microdiscectomy, whereas patients presenting with spinal stenosis (SS) undergo spinal decompression surgery. It should be noted that there are variations of spinal decompression technique—surgeon might choose among different approaches, i.e., interlaminectomy, hemilaminectomy, laminectomy, and others, depending on the degree and levels of the stenosis, as well as the desired decompression of neural structures.

As previously stated, being overweight or obese increases biomechanical stress on the spinal column, which is pronounced in the lower back. Although the exact mechanism is still unknown, prevalence of DH is correlated with increased BMI [7]. Similarly, overweight and obese individuals are at increased risk of developing SS, which is also known as degenerative spine disease [8]. Another factor that is strongly linked with lumbar SS is increased age [15]. The likely explanation of this correlation includes various structural changes that occur within the spinal column during senescence [16]. On the other hand, although supporting research is not as abundant, smoking and genetics also appear to play a role in lumbosacral spine disease [17,18]. Admittedly, none of these factors has been shown to be causal to spine disease, and these factors often interact, which further corroborates the idea of multifactorial etiology.

Not only has the pandemic altered our daily lives, it has also transformed medical practices around the world. In the initial period of the pandemic, the majority of medical attention was on treating patients diagnosed with COVID-19. This in turn affected patients suffering from other diseases. In particular, surgical specialties consistently reported decreases in quantity of elective and emergency procedures during an earlier period of the pandemic which was likely correlated with general public health measures [19,20]. At the same time, there was an increase in telemedicine utilization in surgical practice [21]. However, the data of elective spine surgeries in this specific period are still growing. Several authors have described their observations of neurosurgical practice adaptations—these studies reported declines in elective spinal procedures [22,23,24,25,26]. In this study, we seek to investigate the trends in volume of elective lumbosacral spine surgery prior to the pandemic and during the pandemic. Additionally, we aim to assess the prevalence of common risk factors of patients undergoing elective spine surgery at a single tertiary center during the early post-pandemic period.

## 2. Materials and Methods

This study was performed at the Department of Neurosurgery, University Hospital Center Osijek in Croatia. The total number of elective lumbosacral spine surgeries performed due to DH or SS during 2019, 2020, and 2021 was queried from the surgical logbook of the Department. Average monthly procedures for each year were then calculated. Detailed demographic data of patients who underwent elective lumbosacral spine surgery during 2021 were prospectively collected. We included only patients with DH and/or SS as indications for surgery. Microdiscectomy was performed for patients diagnosed with DH, and spine decompression surgery (interlaminectomy, hemilaminectomy, or laminectomy) was the procedure of choice for patients suffering from SS. Multilevel surgeries without fusion were also included in the analysis. Patients requiring fusion or surgery due to acute spinal trauma, infection, or tumors were excluded from the study. The research protocol was approved by the Ethics Committee of University Hospital Center Osijek (R2-7990/2021, date of approval: 8 June 2021). In order to reduce bias, patients undergoing two or more surgeries during 2021 were included only once in a patient cohort for evaluation of common risk factors. Patients with incomplete data who underwent surgery in 2021 were excluded from the study.

Sex, age, comorbidities, and smoking habits of patients undergoing surgery during 2021 were collected via interviews and from their medical history. Variables needed to calculate BMI, i.e., height and weight, were acquired from preoperative anesthesia evaluations, which were performed at most 30 days prior to the surgery. Diagnosis of spinal pathology was determined by one of seven neurosurgeons from our department based on clinical presentation, accompanying radiologic imaging and intraoperative findings. Consequently, some patients were diagnosed with both DH and SS. In order to evaluate the impact of BMI and age on spinal pathologies within our study sample, patients were divided into classes as follows. BMI was calculated by the standard definition—weight in kilograms divided by height in meters squared. In accordance with WHO classification, based on their BMI values, patients were divided into two weight classes: normal weight with BMI values less than 25 kg/m^2^ and overweight with BMI values of at least 25 kg/m^2^. Due to the fact that there is no standard classification of age groups, we utilized the common stratification we observed in other studies. Based on their age, patients were stratified into two groups: the adult group, which consisted of patients younger than 65 years of age, and the elderly, which included patients who were at least 65.

Continuous variables were summarized as means and standard deviation and compared using one-way ANOVA with post-hoc Tukey HSD test. Variables which are not normally distributed were analyzed using Mann–Whitney U-test. Categorical variables were summarized as frequencies and proportions and compared using the χ^2^ test with Bonferroni correction when applicable. All *p* values were two-sided, and the threshold for statistical significance was *p* ≤ 0.05.

## 3. Results

### 3.1. Number of Procedures

**Figure 1:**
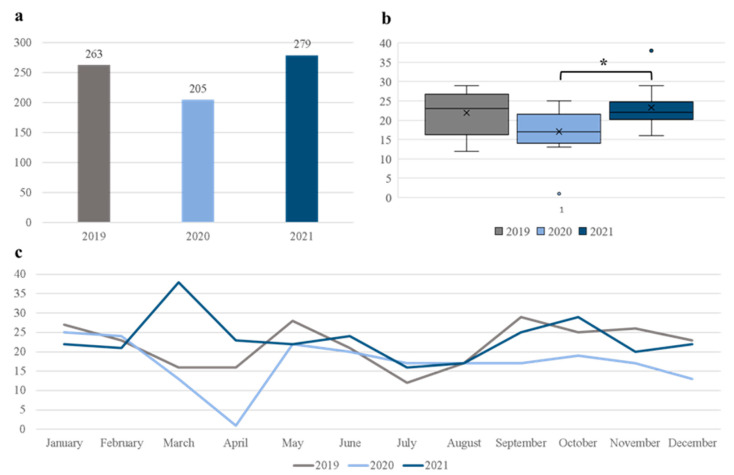
Cumulative number of elective lumbosacral spine surgeries before and during the pandemic (**a**), average monthly procedures for a given year (**b**), and the total number of surgeries per month for the study period (**c**). * *p* < 0.05, one-way ANOVA with post-hoc Tukey HSD.

In the three-year period from the start of 2019 till the end of 2021, a total of 747 elective lumbosacral spine procedures due to DH or SS were performed at the Department of Neurosurgery, University Hospital Center Osijek. The number of surgeries during 2020, the first year of the COVID-19 pandemic in our country, decreased by 22.1% compared to the prepandemic year of 2019. Furthermore, the number of procedures performed at our department throughout 2021 increased by 36.1% compared to 2020. These results are depicted visually in Figure 1.

In order to better understand the differences in surgical volume during the study period, we calculated the average number of surgeries performed in a month, which is presented in Figure 1b. The average number of monthly procedures for 2019, 2020, and 2021 were 21.9 ± 5.5, 17.1 ± 6.3, and 23.3 ± 5.8, respectively. There was a statistically significant difference between these time-periods as determined by one-way ANOVA (F (2, 33) = 3.66, *p* < 0.05). The post hoc Tukey HSD test revealed significant increase in the average monthly procedures for 2021 compared to 2020 (*p* = 0.039). Trends in monthly surgeries during the study period are presented in Figure 1c.

### 3.2. Characteristics of Patients in 2021

Throughout 2021, there was a total of 242 patients who satisfied the inclusion criteria. Patient characteristics are summarized in Table 1. In the sample, both men and women were equally represented (121 men and 121 women). The youngest patient was 19 years old, whereas the oldest was 81. The average age of patients who underwent elective lumbosacral spine surgery at our department throughout 2021 was 53 years (53.14 ± 13.05). The mean BMI of our patients was 28.3 kg/m^2^ (28.31 ± 4.89 kg/m^2^). The lowest recorded BMI was 18.07 kg/m^2^ and the highest was 46.88 kg/m^2^. Regarding smoking status, 103 (42.6%) patients were smokers. According to their BMI values, patients were grouped into two weight classes in accordance with WHO classification [12]. There were 63 (26%) patients with BMI less than 25 kg/m^2^, and as such were characterized as having normal weight, whereas 179 (74%) patients had BMI of at least 25 kg/m^2^ and were characterized as overweight (Table 1). These two groups did not significantly differ in terms of age or the number of smokers in the group. In order to further describe the correlation of weight status and sex, χ^2^ test of independence was performed which was significant: χ^2^ (1, N = 242) = 6.2, *p* = 0.013.

Out of 242 patients, 107 (44.2%) did not have any comorbidities. Of all the documented comorbidities, the most common was hypertension (42.1%), followed by diabetes (9.9%) and gastrointestinal disorders (7.0%). The full list of reported comorbidities is presented in Table 2.

### 3.3. Spine Pathology Analysis

In order to examine the association of spine pathologies with other variables, i.e., age group, weight class, and sex, we utilized χ^2^ tests with Bonferroni correction. The results are presented in Table 3. The association between DH and age group was significant: χ^2^ (1, N = 242) = 28.7, *p* < 0.001. Patients who were younger than 65 years were more likely to undergo surgery due to DH. Similarly, association between SS and age group was also significant: χ^2^ (1, N = 242) = 26.2, *p* < 0.001. This implies that elderly patients (those who were at least 65 years of age) in our sample were more likely to present with SS. However, we did not detect any significant association between spine pathology, weight class, and sex.

## 4. Discussion

In this study, we described the trends in elective lumbosacral spine surgery prior to the COVID-19 pandemic, and during the first two years of the pandemic. We observed a decrease of 22.1% in the number of procedures in the initial year of the pandemic compared to the year before (263 surgeries in 2019 and 205 surgeries in 2020). This finding is in line with other contemporary studies that reported a decrease in other elective surgeries in these unique circumstances [19,20,22,23,24,25,26]. The described reduction in surgical volume is most likely a direct consequence of reorganization of healthcare in order to better treat patients with COVID-19, but at the same time to limit the spread of SARS-CoV-2 in hospitals. Simultaneously, surgical specialties adapted and increased the utilization of telemedicine in treatments of their patients [21,25], with the same goal of limiting exposure to the virus. In contrast, in our study, the number of elective procedures during the second year of the pandemic increased by 36.1% when compared to the initial pandemic year (205 surgeries in 2020 and 279 surgeries in 2021). Additionally, the average number of surgeries per month was significantly higher in 2021 compared to 2020. The most striking reduction in surgical volume for 2020 was observed in March and April (Figure 1c), as those were the months with the most pronounced public health policies. During this period, most of the elective surgeries in our country were postponed due to the rapid spreading of the virus. The public health measures were implemented by the government during 2020, which was reflected in the reduction of the total number of procedures. Healthcare workers in our hospital, and including our department, were reassigned to treat patients diagnosed with COVID-19. Another reason for this reduction could be attributed to some individual patients’ decision to postpone the surgery if possible due to the fear of being infected with the virus. However, this temporary reduction and postponement resulted in a backlog of surgeries, which was reflected in the following year. Interestingly, Mills et al. [24] described a similar trends in spine surgery during their study period. One explanation for this finding could be the propensity towards sedentary lifestyle in the era of social distancing and other epidemiological measures enforced in order to reduce the spread of SARS-CoV-2 [1,27]. A higher incidence of LBP in this same period further supports this hypothesis [3]. Another explanation for this result is the general delay of elective surgeries at the start of the pandemic [28,29,30]. As it was speculated at the start of the first COVID-19 wave, cancellations and postponements of elective surgeries would eventually result in compensatory increase in surgical volume when the epidemiological measures were not as strict, with the aim of clearing the backlog of surgeries from the COVID-19 pandemic [30]. Taken together, the rise in surgeries in our department is likely explained by many factors. Further prospective studies are needed to better address this question and to verify the increased incidence of spine-related problems, such as LBP, and propensity towards sedentary lifestyle.

As mentioned above, in our cohort, both men and women were equally represented. It should be noted that more than three quarters of our patients were overweight or obese. In comparison, it was estimated that the prevalence of overweight and obesity in Croatia in 2015 was 58.2% [31]. Our cohort had a greater proportion of individuals with increased BMI when compared to the general population. This finding is in line with other previously published research, suggesting that being overweight is a major risk factor for DH, as well as SS [7,8,9,10], even at the time of the pandemic. Furthermore, overweight and obese individuals have an increased risk for other metabolic diseases, most notably hypertension and type 2 diabetes [32,33,34]. Metabolic syndrome is a complex condition consisting of several metabolic disturbances: hypertension, diabetes, and obesity [34]. As previously stated, hypertension and diabetes were the most prevalent comorbidities in our sample. Severe LBP in most cases decreases physical activity and, consequently, energy expenditure, thereby increasing weight gain and BMI. If this state persists for longer periods, it might also increase vulnerability to the aforementioned metabolic disturbances. However, these interactions are probably bidirectional—patients suffering from metabolic diseases are more likely to eventually suffer from LBP due to their sedentary lifestyle (reduced physical activity) and other contributing factors. Taken together, this suggests that LBP might be an underappreciated component of metabolic syndrome which significantly aggravates the wellbeing of patients suffering from this chronic condition—and this was further exacerbated during the COVID-19 pandemic.

Furthermore, we wanted to understand if one sex was more vulnerable to previously described spinal pathologies, while taking weight class into consideration. Interestingly, the association of weight class and sex was significant. Patients with normal weight who underwent elective spine surgery in our department were more likely to be women, whereas overweight patients were mostly men. It should be noted that the prevalence of overweight in Croatia is greater for men than for women, which likely influenced our results [31]. Despite the disparity of overweight patients, both sexes were equally represented in our cohort, and sex-specific structural and biomechanical differences in spine anatomy should be appreciated [35]. We also found an age-group-related distribution of spinal pathologies—DH was more prevalent in patients younger than 65 years of age, whereas SS was more prevalent in older patients. A possible explanation for this finding was first suggested by Jönnson and Strömqvist [16]. They proposed that during senescence, structural changes, such as ligamentum flavum thickening and facet hypertrophy, happen within the lumbosacral spine and this gradually shifts the clinical presentation of DH, as well as radiological findings, towards SS. Of these age-related structural changes, ligamentum flavum thickening and facet hypertrophy contribute the most to narrowing of the spinal canal.

### Limitations

There are some important limitations to our study. Our study focused on the specific population of patients in Eastern Croatia and, combined with relatively small sample size, cannot be generalized to other populations. It would be interesting to replicate our study in other countries for the same period in order to gain better insights into elective spine surgery trends. A major pitfall of our study is that we only collected demographic data for patients undergoing surgery during 2021, as we were not able to retrieve the same variables for the prior period. Furthermore, although sedentary lifestyle likely played a major role in the increase of spine surgeries in 2021, we did not specifically test this hypothesis with questionnaires. Moreover, due to the small sample size, we were not able to control specific factors and indices, which could potentially bias our results.

## 5. Conclusions

In the analysis of elective lumbosacral spine surgery prior to and during the pandemic, we observed an initial decrease in the number of surgeries which was followed by a compensatory increase the following year. Overweight and obesity prevalence is reaching epidemic status, which presents a major medical and economic challenge worldwide [12]. Having increased BMI increases vulnerability to other metabolic diseases, primarily hypertension and diabetes. However, being overweight or obese also increases susceptibility to LBP, one of the leading causes of disability. Patients suffering from debilitating chronic LBP are likely to seek medical attention, and in specific cases are often candidates for surgical treatment—either microdiscectomy if they suffer from DH or spinal canal decompression if they suffer from SS. This was all reflected in our results.

This study provides insights into neurosurgical data of Croatian patients undergoing lumbosacral spine surgery in the unique circumstances of the COVID-19 pandemic. Delays of elective procedures at the start of the pandemic will likely increase the surgical volume in the near future, which was evident from our data. Furthermore, sedentary lifestyle, in addition to having metabolic consequences, seems to have important biomechanical repercussions in the lumbosacral spine, which was accentuated during the period of epidemiologic policies.

## Figures and Tables

**Table 1:** Characteristics of patients who underwent elective lumbosacral spine surgery in 2021.

Variables	Normal Weight	Overweight	Total	*p* Value
**Patients**	63 (26%)	179 (74%)	242	
**BMI (kg/m^2^)**	22.75 ± 1.83	30.14 ± 4.11	28.31 ± 4.89	
**Smokers**	32 (50.8%)	71 (39.7%)	103 (42.6%)	0.12
**Age (years)**	52.30 ± 13.2.3	53.43 ± 13.00	53.14 ± 13.05	0.66
**Sex**				
**Male**	23 (36.5%)	98 (54.7%)	121	0.013 *
**Female**	40 (63.5%)	81 (45.3%)	121	

BMI: body mass index. * *p* < 0.05, chi-square test with Bonferroni correction.

**Table 2:** Comorbidities of patients who underwent elective lumbosacral spine surgery in 2021.

Comorbidity	Number of Patients
None	107 (44.2%)
Hypertension	102 (42.1%)
Diabetes	24 (9.9%)
Gastrointestinal disorders	17 (7.0%)
Thyroid disorders	9 (3.7%)
Respiratory disorders	5 (2.1%)
Cardiac disorders	5 (2.1%)
Autoimmune disorders	5 (2.1%)
Hematologic disorders	4 (1.7%)
Miscellaneous	4 (1.7%)

**Table 3:** The associations of the spinal pathology with age group, weight class, and sex.

	Number of Patients	Spine Pathology	
		Disc Herniation	Spinal Stenosis
**Adults (18–64)**	195	143 (73.3%) *	55 (28.2%)
**Elderly (65+)**	47	15 (31.9%)	32 (68.1%) *
**BMI < 25 kg/m²**	63	44 (69.8%)	20 (31.7%)
**BMI ≥ 25 kg/m^2^**	179	114 (63.7%)	67 (37.4%)
**Male**	121	79 (65.3%)	44 (36.4%)
**Female**	121	79 (65.3%)	43 (35.5%)

BMI: body mass index. * *p* < 0.001, χ^2^ test with Bonferroni correction.

## Data Availability

Data will be available upon request.

## References

[1] Wunsch K., Kienberger K., Niessner C. (2022). Changes in Physical Activity Patterns Due to the COVID-19 Pandemic: A Systematic Review and Meta-Analysis. Int. J. Environ. Res. Public Health.

[2] Mignogna C., Costanzo S., Ghulam A., Cerletti C., Donati M.B., de Gaetano G., Iacoviello L., Bonaccio M. (2021). Impact of Nationwide Lockdowns Resulting from the First Wave of the COVID-19 Pandemic on Food Intake, Eating Behaviors, and Diet Quality: A Systematic Review. Adv. Nutr..

[3] Papalia G.F., Petrucci G., Russo F., Ambrosio L., Vadalà G., Iavicoli S., Papalia R., Denaro V. (2022). COVID-19 Pandemic Increases the Impact of Low Back Pain: A Systematic Review and Metanalysis. Int. J. Environ. Res. Public Health.

[4] Edwards J., Hayden J., Asbridge M., Gregoire B., Magee K. (2017). Prevalence of low back pain in emergency settings: A systematic review and meta-analysis. BMC Musculoskelet. Disord..

[5] Vos T., Allen C., Arora M., Barber R.M., Bhutta Z.A., Brown A., Carter A., Casey D.C., Charlson F.J., Chen A.Z. (2016). Global, regional, and national incidence, prevalence, and years lived with disability for 310 diseases and injuries, 1990-2015: A systematic analysis for the Global Burden of Disease Study 2015. Lancet.

[6] Hartvigsen J., Hancock M.J., Kongsted A., Louw Q., Ferreira M.L., Genevay S., Hoy D., Karppinen J., Pransky G., Sieper J. (2018). What low back pain is and why we need to pay attention. Lancet.

[7] Sheng B., Feng C., Zhang D., Spitler H., Shi L. (2017). Associations between Obesity and Spinal Diseases: A Medical Expenditure Panel Study Analysis. Int. J. Environ. Res. Public Health.

[8] Knutsson B., Sandén B., Sjödén G., Järvholm B., Michaëlsson K. (2015). Body Mass Index and Risk for Clinical Lumbar Spinal Stenosis: A Cohort Study. Spine.

[9] Frilander H., Solovieva S., Mutanen P., Pihlajamäki H., Heliövaara M., Viikari-Juntura E. (2015). Role of overweight and obesity in low back disorders among men: A longitudinal study with a life course approach. BMJ Open.

[10] Nuttall F.Q. (2015). Body Mass Index: Obesity, BMI, and Health: A Critical Review. Nutr. Today.

[11] Prentice A.M., Jebb S.A. (2001). Beyond body mass index. Obes. Rev..

[12] World Health Organization (2000). Obesity: Preventing and managing the global epidemic. Report of a WHO Consultation.

[13] Foster N.E., Anema J.R., Cherkin D., Chou R., Cohen S.P., Gross D.P., Ferreira P.H., Fritz J.M., Koes B.W., Peul W. (2018). Prevention and treatment of low back pain: Evidence, challenges, and promising directions. Lancet.

[14] National Guideline Centre (2016). National Guideline Centre. National Institute for Health and Care Excellence: Guidelines. Low Back Pain and Sciatica in Over 16s: Assessment and Management.

[15] Kolte V.S., Khambatta S., Ambiye M.V. (2015). Thickness of the ligamentum flavum: Correlation with age and its asymmetry-an magnetic resonance imaging study. Asian Spine J..

[16] Jönsson B., Strömqvist B. (1995). Influence of age on symptoms and signs in lumbar disc herniation. Eur. Spine J..

[17] Elmasry S., Asfour S., de Rivero Vaccari J.P., Travascio F. (2015). Effects of Tobacco Smoking on the Degeneration of the Intervertebral Disc: A Finite Element Study. PLoS ONE.

[18] Colombini A., Brayda-Bruno M., Ferino L., Lombardi G., Maione V., Banfi G., Cauci S. (2015). Gender differences in the VDR-FokI polymorphism and conventional non-genetic risk factors in association with lumbar spine pathologies in an Italian case-control study. Int. J. Mol. Sci..

[19] Mattingly A.S., Rose L., Eddington H.S., Trickey A.W., Cullen M.R., Morris A.M., Wren S.M. (2021). Trends in US Surgical Procedures and Health Care System Response to Policies Curtailing Elective Surgical Operations During the COVID-19 Pandemic. JAMA Netw. Open.

[20] Sá A.F., Lourenço S.F., Teixeira R.D.S., Barros F., Costa A., Lemos P. (2021). Urgent/emergency surgery during COVID-19 state of emergency in Portugal: A retrospective and observational study. Braz. J. Anesthesiol..

[21] Koruga N., Koruga A.S., Rončević R., Turk T., Kopačin V., Kretić D., Rotim T., Rončević A. (2022). Telemedicine in Neurosurgical Trauma during the COVID-19 Pandemic: A Single-Center Experience. Diagnostics.

[22] Wordie S.J., Tsirikos A.I. (2021). The impact of the COVID-19 pandemic on spinal surgery. Orthop. Trauma.

[23] Attaripour B., Xiang S., Mitchell B., Siow M., Parekh J., Shahidi B. (2022). A Retrospective Study of the Impact of COVID-19 Pandemic Related Administrative Restrictions on Spine Surgery Practice and Outcomes in an Urban Healthcare System. Int. J. Environ. Res. Public Health.

[24] Mills E.S., Mertz K., Faye E., Ton A., Wang J.C., Hah R.J., Alluri R.K. (2023). The Effect of COVID-19 on Spine Surgery. Glob. Spine J..

[25] Lucke-Wold B., Cerillo J.L., Becsey A.N., Chernicki B.P., Root K.T. (2022). Minimally Invasive Procedures, Perioperative Telemedicine, and Decreased Hospital Stays Following COVID-19 Surgical Restrictions: Spinal Surgery. Arch. Med. Case Rep. Case Study.

[26] Lin G.X., Kotheeranurak V., Chen C.M., Hu B.S., Rui G. (2022). Global research hotspots and trends in the field of spine surgery during the COVID-19 pandemic: A bibliometric and visual analysis. Front. Surg..

[27] Lin J.A., Braun H.J., Schwab M.E., Pierce L., Sosa J.A., Wick E.C. (2021). Pandemic Recovery: Persistent Disparities in Access to Elective Surgical Procedures. Ann. Surg..

[28] Jain A., Jain P., Aggarwal S. (2020). SARS-CoV-2 Impact on Elective Orthopaedic Surgery: Implications for Post-Pandemic Recovery. The Journal of bone and joint surgery. J. Bone Joint. Surg. Am..

[29] Saggaf M., Anastakis D. (2021). The Impact of COVID-19 on the Surgical Wait Times for Plastic and Reconstructive Surgery in Ontario. Plast. Surg..

[30] Collaborative C. (2020). Elective surgery cancellations due to the COVID-19 pandemic: Global predictive modelling to inform surgical recovery plans. Br. J. Surg..

[31] Gallus S., Lugo A., Murisic B., Bosetti C., Boffetta P., La Vecchia C. (2015). Overweight and obesity in 16 European countries. Eur. J. Nutr..

[32] Koliaki C., Liatis S., Kokkinos A. (2019). Obesity and cardiovascular disease: Revisiting an old relationship. Metabolism.

[33] Patel S.A., Ali M.K., Alam D., Yan L.L., Levitt N.S., Bernabe-Ortiz A., Checkley W., Wu Y., Irazola V., Gutierrez L. (2016). Obesity and its Relation with Diabetes and Hypertension: A Cross-Sectional Study Across 4 Geographical Regions. Glob. Heart.

[34] Eckel R.H., Grundy S.M., Zimmet P.Z. (2005). The metabolic syndrome. Lancet.

[35] Mohan M., Huynh L. (2019). Sex Differences in the Spine. Curr. Phys. Med. Rehabil. Rep..

